# Role of Natural Products in Ameliorating Drugs and Chemicals Toxicity

**DOI:** 10.1155/2016/7879406

**Published:** 2016-08-23

**Authors:** Mohamed M. Abdel-Daim, Salah M. Aly, Khaled Abo-el-Sooud, Mario Giorgi, Sorin Ursoniu

**Affiliations:** ^1^Pharmacology Department, Faculty of Veterinary Medicine, Suez Canal University, Ismailia 41522, Egypt; ^2^Pathology Department, Faculty of Veterinary Medicine, Suez Canal University, Ismailia 41522, Egypt; ^3^Pharmacology Department, Faculty of Veterinary Medicine, Cairo University, Giza, Egypt; ^4^Pharmacology and Toxicology Division, Department of Veterinary Sciences, University of Pisa, Pisa, Italy; ^5^Department of Functional Sciences, Discipline of Public Health, “Victor Babes” University of Medicine and Pharmacy, Timisoara, Romania

Herbal medicines have a long history over than 7000 years in traditional treatment, therapeutic experiences, and clinical trials including Egypt, China, and Korea. This practice is still the mainstay of about 75–80% of the world population, mainly in the developing countries, for primary health care and promotion because of better cultural acceptability, better compatibility with the human body, and lesser side effects. However, the last few years have seen a major increase in their use in the developed world. Nowadays, we can find a bipolarised market for the active ingredients: those chemically produced and mainly supported by the pharmaceutical companies and those natural constituents that are demanded by an increased number of patients. Although natural products have not been always active as supposed, some of them are scientifically recognised as therapeutically active. Indeed, it has to be noted that some drugs, still used in the current therapies, are extracted from plants. Some of these can have additive action if coadministered with synthesized drugs or ameliorate the drug toxicity.

Potentially there are hundred thousands of natural compounds on the heart. Countries where the flora and fauna are variegated have more potentiality. That is the reason why research on natural compound is especially developed in these areas. However, the increased global demand of these active ingredients has led to a worldwide research in this field. The reason why this special issue has been set up is because the natural compounds are taking place in our society, and their combination with synthesized drugs is an “on the edge” topic.

Nine manuscripts have been published.

L.-J. Cao et al. concluded that isoliquiritigenin and glycyrrhetinic acid could activate the nuclear factor erythroid 2-related factor 2 (Nrf2) antioxidant response in HepG2 cells, protecting against triptolide-induced oxidative damage.

X. Jing et al. suggest that electroacupuncture is an effective approach for inhibiting weight gain in type 2 diabetic rats treated by rosiglitazone through increased levels of leptin receptor and STAT3 and decreased PPAR*γ* expression.

S. Paul et al. examined the possible protective effects of Satkara,* Citrus macroptera* fruit ethanol extract against acetaminophen-induced rats hepatorenal toxicity through its inhibition of lipid peroxidation.

A. Hanafy et al. evaluated the hepatoprotective and antioxidant activities of the methanol extract of* Adansonia digitata* fruit pulp on acetaminophen-induced hepatotoxicity in rats.

Y. Luo et al. conducted a randomized, parallel-group, multicenter clinical application study in order to confirm the Kangfuxin solution superiority to compound borax gargle on chemoradiotherapy-mucositis.

D. L. Valle Jr. et al. isolated and identified the antimicrobial compounds of Philippine* Piper betle* L. leaf ethanol extracts by thin layer chromatography- (TLC-) bioautography and gas chromatography-mass spectrometry (GC-MS) and tested them against two Gram-positive multidrug-resistant (MDR) bacteria.

N. H. AbdelAllah et al. used an alternative natural adjuvant system (chitosan and sodium alginate) allowing for a reduction in dose and cost in the antihepatitis B vaccine.

I. J. Asiedu-Gyekye et al. investigated the elemental composition of unsweetened natural cocoa powder, its effect on nitric oxide, and its hepatoprotective potential during simultaneous administration with high-dose artemether/lumefantrine (recommended therapy for malaria). They found that unsweetened natural cocoa powder increases nitric oxide levels and has hepatoprotective potential during artemether/lumefantrine administration.

S. Sabiu et al. evaluated membrane stabilization and detoxification potential of ethyl acetate fraction of* Zea mays* L.,* Stigma maydis* in acetaminophen-induced renal oxidative damage through its antioxidant effect.

*Mohamed M. Abdel-Daim* *Mohamed M. Abdel-Daim* *Salah M. Aly* *Salah M. Aly* *Khaled Abo-el-Sooud* *Khaled Abo-el-Sooud* *Mario Giorgi* *Mario Giorgi* *Sorin Ursoniu* *Sorin Ursoniu*

